# Modeling Terror Attacks with Self-Exciting Point Processes and Forecasting the Number of Terror Events

**DOI:** 10.3390/e25071011

**Published:** 2023-06-30

**Authors:** Siyi Wang, Xu Wang, Chenlong Li

**Affiliations:** 1 Department of Mathematics, Wilfrid Laurier University, Waterloo, ON N2L 3C5, Canada; wangs424@mcmaster.ca; 2 College of Mathematics, Taiyuan University of Technology, Taiyuan 030024, China; lichenlong@tyut.edu.cn

**Keywords:** self-exciting point process, Hawkes process, terrorism, random forests

## Abstract

Rampant terrorism poses a serious threat to the national security of many countries worldwide, particularly due to separatism and extreme nationalism. This paper focuses on the development and application of a temporal self-exciting point process model to the terror data of three countries: the US, Turkey, and the Philippines. To account for occurrences with the same time-stamp, this paper introduces the order mark and reward term in parameter selection. The reward term considers the triggering effect between events in the same time-stamp but different order. Additionally, this paper provides comparisons between the self-exciting models generated by day-based and month-based arrival times. Another highlight of this paper is the development of a model to predict the number of terror events using a combination of simulation and machine learning, specifically the random forest method, to achieve better predictions. This research offers an insightful approach to discover terror event patterns and forecast future occurrences of terror events, which may have practical application towards national security strategies.

## 1. Introduction

The number of countries that suffer from terrorist attacks has shown a general increasing trend over the past decade. The global death number in 2010 due to terrorism was about 8000, reaching as high as 44,000 in 2014. Terrorist attacks caused the fatality of least one person in 79 countries in 2016 [1]. Terrorism has become a non-negligible problem for many countries, and it is meaningful to analyze terror attack patterns and the latent risks of terrorism.

The systematic collection of terrorism data has prompted researchers to use criminological and sociological theories to understand its roots and devise prevention strategies [2]. Examples of these theories include Relative Deprivation Theory (RDT), General Strain Theory (GST) and Social Learning Theory (SLT). RDT posits that perceived inequalities among groups may foster terrorism, especially among oppressed underclasses, while GST suggests that unjust treatment by powerful entities could incite terrorism [2]. However, a direct link between deprivation, strains, and terrorism is yet to be fully substantiated, limiting these theories’ predictive capabilities. Akins and Winfree [3] presented the utility of Social Structure Social Learning (SSSL), an expanded version of SLT, in terrorism studies. This model treats terrorism as a learned behavior, providing a new lens for investigating the mechanisms of terrorist groups, including their recruitment methods. They proposed that radicalization should be viewed as a social learning process, which emphasizes the role of social networks in spreading radical ideas and encouraging self-radicalization. Thus, under this framework, terrorist activities can be understood not as isolated incidents but as outcomes influenced by the ongoing ripple effects of existing terrorist organizations and past events.

Following the descriptions in the SSSL model, radicalization may be understood as individuals being affected with extremist ideologies from contacts with terrorist organizations or actions. It lends itself to the interpretation that the spread of some terror events is the result of contagious effects, where one terror incident could incite subsequent attacks. Some studies have indeed shown that terrorism events tend to be contagious. For example, Midlarsky el al. [4] worked on the contagion of terrorism throughout the world, and they discovered that terror attacks are contagious not only within the same terrorist organization but also among different groups or even countries.

An effective statistical model can be illuminating in understanding patterns and trends of terror attacks. Where the events are temporally clustered within a continuous time domain, a temporal self-exciting point process could serve as an appropriate statistical model. In the research of Townsley et al. [5] analyzing the terror data of Iraq, the authors showed that even unrelated terror events were clustered in space and time. Other research in Israel [6] also found that the risk of a subsequent terror attack increases significantly on the day following an attack. These works provide the justification of applying self-exciting point processes on terror data. Later, Levis et al. [7], Mohler et al. [8], Khraibani [9] and Clark et al. [10] all developed models based on self-exciting point processes for analyzing terror or crime data. Furthermore, there have been studies providing more theoretical foundations for the phenomenon of self-excitation observed in terror attacks. Clauset and Gleditsch’s work [11] is a notable example, where they examined the feedback loop present in terrorist organizations. This feedback loop suggests that new attacks result in organizational growth, which in turn leads to an acceleration in the production of subsequent events.

**Figure 1:**
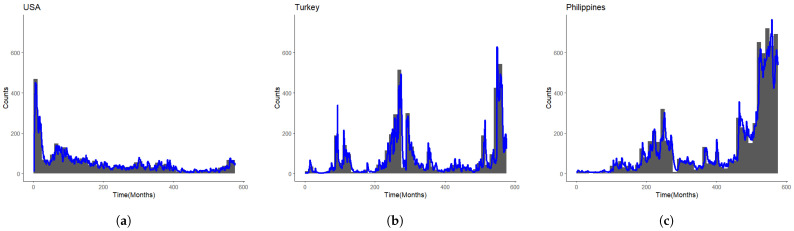
Fitting results with the month-based arrival times of the self-exciting process with the KDE-based smooth background rate in three representative countries. The histograms with 48 bins in each graph represent the true number of events, while the blue lines stand for the estimated intensity in the same scale: (**a**) The true and estimated number of terror events of the US from 1970 to 2017. (**b**) The true and estimated number of terror events of Turkey from 1970 to 2017. (**c**) The true and estimated number of terror events of the Philippines from 1970 to 2017.

This research uses the terror data from the Global Terrorism Database (GTD), supported by the University of Maryland. GTD contains data of terror attacks from 1970 to 2018 for 205 countries. We used the *K*-means algorithm to cluster these countries in the GTD data set; then, we selected three representative countries exhibiting distinct temporal patterns of terror attacks. These three countries are the United State of America (the US), Turkey and the Philippines. The US experienced a peak of terror attacks in the last century, while the annual counts of attacks have remained relatively steady over the past decade. Turkey, on the other hand, demonstrated noticeable clustering patterns of terror events, especially with three pronounced peaks occurring during the period from 1970 to 2018. Meanwhile, the majority of terror attacks in the Philippines have been concentrated in recent years (see Figure 1). Furthermore, these countries also present unique geographical features. The Philippines, an island nation in Southeast Asia, contrasts with Turkey, situated at the junction of Western Asia and Europe. These countries, along with the US, have been frequently studied in the context of terror attacks [12,13,14].

Even though numerous approaches based on self-exciting point processes have been developed to model and analyze terror data, there remains a dearth of research on employing these models to forecast short-term trends of terror attacks. Traditional prediction strategies for temporal point processes, such as those mentioned by Yang [15], either aggregate the intensities within a specified time window or simulate the event arrival times within that duration. Our study introduces a novel forecasting framework that combines traditional simulation methods with machine learning techniques, specifically the random forest algorithm. This combination seeks to enhance the accuracy of predicting the count of terror events in the immediate future, namely, the upcoming year.

To assess the prediction performance of the number of events, the data from three sample countries are divided into training and test sets, using the data from 1970 to 2017 as the training sets and the data from 2018 as test sets. It is necessary to note that although a temporal point process can be used to model the occurrence of events without imposing a predetermined time window, real-world data usually possess a limited level of granularity in terms of time-stamp. For instance, it is possible for two events to transpire on the same day but at disparate times, with the database only registering the date of occurrence. Although the majority of records in the GTD database include the year, month, and date of events, some entries may be missing the exact date information. In this study, based on our investigation, we propose a model using months as the unit of time. This simplifies the model and greatly alleviates the computational burden associated with parameter estimation and simulation. Later, this month-based model is compared with a day-based model to further verity its superior performance. To fit the data, a self-exciting model with a flexible smooth background intensity function proposed by Lewis [7] is adaptively modified to explore our data. A nonparametric method called Gaussian Kernel Density Estimation (KDE) is used to estimate the smooth background intensity function. In summary, two main innovations are proposed in this research: (1) Lewis’s model is carefully modified and fine-tuned for our data, especially addressing the issue of multiple events being recorded under the same time-stamp due to the data being recorded on a monthly basis. The key is the introduction of two components in the model: the order marker and the reward term. (2) A new algorithm that combines both simulation and machine learning methods is developed so that the number of events in a short time window can be predicted with higher accuracy.

The rest of this paper is organized as follows. Section 2 explains in detail the framework and the parameter estimation of the self-exciting point process with a smooth background intensity function. The results of the self-exciting model in three sample countries are provided in Section 3. Section 4 describes the thinning method for simulation, then discusses the approaches for predicting the number of terror attacks in a year. The paper is concluded with discussions and the future directions in Section 5.

## 2. Self-Exciting Point Processes

### 2.1. Hawkes Process

A well-known self exciting process was proposed by Alan G. Hawkes in 1971 and has subsequently been called the “Hawkes process” [16]. A conditional intensity function of the Hawkes process consists of an exogenous intensity that describes the background factors and an endogenous intensity which represents the triggering effects of previous events [17]. The conditional intensity function of a one-dimensional Hawkes process has a basic form as in (1).

(1) $$\begin{array}{c} \lambda \left(t|H\left(t\right)\right)=\lambda_{0}\left(t\right)+\sum_{t_{i}<t}Y\left(t-t_{i}\right), \end{array}$$

where H(t) is the history at time *t*, $\lambda_{0}\left(t\right)$ is a deterministic background intensity and $t_{i}$ in the excitation function $\sum_{t_{i}<t}Y\left(t-t_{i}\right)$ represents the arrival time of the *i*th event. The excitation function is often taken to be an exponential function of time [18]. For convenience, we write λ(t|H(t)) as λ(t) instead. An alternative view of the Hawkes process as represented by (1) is a Poisson cluster process as in [19]. All generated events of the Hawkes process can be separated into two categories:

- Immigrants: The events arrive independently in a system, i.e., events are described by the exogenous intensity.
- Offspring: The events are triggered by previous existing events, i.e., events are described by the endogenous intensity.

Thus, immigrants generate independent clusters. The offspring are structured into those clusters, where the center of each cluster is an immigrant event. This is called the branching structure. The expected number of offspring generated by each single event is called “branching ratio”, which is denoted by $\eta =\int_{0}^{\infty }Y\left(t\right)dt$, where Y(t) is the excitation term in (1). If η<1, the process is subcritical, and the total number of events in each cluster is finite. If η>1, the process is supercritical, and the total number of events in each cluster is unbounded [20].

### 2.2. Smooth Background Intensity

In many applications of self-exciting point processes, the background intensity function is assumed to be a constant [16,21]. To model terror attack data, a constant background intensity is not sufficient, because the long-term trend of terrorism has changed significantly over the past decades [1]. Equation (2) gives the conditional intensity function of the self-exciting model proposed by Lewis et al. [7].

(2) $$\begin{array}{c} \lambda \left(t\right)=pn\mu_{sm}\left(t\right)+\left(1-p\right)k_{0}\sum_{t_{i}<t}\omega e^{-\omega \left(t-t_{i}\right)}. \end{array}$$

In this model, the parameter *p* gives the proportion of events attributed to the background intensity. The two positive parameters $k_{0}$ and ω control the arrival jump of the intensity and decay rate of the exponential term, respectively. The smooth background rate $\mu_{sm}\left(t\right)$ based on Gaussian KDE is given by

(3) $$\mu_{sm}\left(t\right)=\frac{1}{n}\sum_{i=1}^{n}\frac{1}{\sqrt{2\pi h_{i}^{2}}}e^{-{\left(t-t_{i}\right)}^{2}/\left(2h_{i}^{2}\right)},$$

where *n* is the total number of events, $t_{i}$ is the event time of the *i*th event and $h_{i}$ is the bandwidth for the *i*th event.

The background intensity function $\mu_{sm}\left(t\right)$ is the average of *n* normal distributions contributed from *n* data points. For each normal distribution, the mean is the recorded time of the corresponding event, and the standard deviation is related to the value of the bandwidth.

The K-Nearest Neighbor (KNN) method, an intuitive choice for determining the bandwidth value, sets $h_{i}$ as the maximum distance between the *i*th event and its *k*th nearest neighbor. The value of *k* is usually proportional to $n^{4/5}$, aiming to balance bias and variance [22], i.e., $k=Cn^{4/5}$, where *C* is a constant. The further constraint of $C<n^{1/5}$ ensures that *k* does not exceed the sample size. The constant *C* is typically determined by minimizing the mean integrated squared error [23]. However, in our model, the background intensity should not be excessively flexible, because overfitting will obscure the contribution from self-excitation. Therefore, we prefer a relatively larger value of *C*. In this study, *C* is chosen within the range of $\frac{n^{1/5}}{3}$ and $\frac{n^{1/5}}{2}$.

### 2.3. Parameter Estimation

Maximum Likelihood Estimation (MLE) can be directly applied to (2) for parameter estimation. The following likelihood function of the Hawkes process is given by Daley and Vere-Jones [24].

**Definition 1**  (Hawkes process likelihood)**.** *Let N(t) be a regular point process on (0,T] with realizations $t_{1},t_{2},\cdots ,t_{n}$. Then, the likelihood function of N(t) is given by*

(4) $$\begin{array}{c} L\left(\vec{\theta }\right):=f\left(t_{1},t_{2},\cdots ,t_{n}\right)=\exp\left(-\int_{0}^{T}\lambda \left(t\right)dt\right)\prod_{i=1}^{n}\lambda \left(t_{i}\right), \end{array}$$

*where $\vec{\theta }$ represents the vector of parameters.*

The log-likelihood function on [0,T] is then

(5) $$\begin{array}{c} L\left(\vec{\theta }\right)=\sum_{i=1}^{n}\log\left(\lambda \left(t_{i}\right)\right)-\int_{0}^{T}\lambda \left(u\right)du. \end{array}$$

The second term $\int_{0}^{T}\lambda \left(u\right)du$ is also called *compensator* Λ(T). We can split the integral [0,T] into disjoint segments $\left[0,t_{1}\right)$, $\left[t_{1},t_{2}\right)$, ⋯, $\left[t_{n-1},T\right]$. Since $\mu_{sm}\left(t\right)$ is a kernel density function, and

$$\int_{0}^{T}pn\mu_{sm}\left(t\right)\approx pn,$$

the compensator Λ(T) of the log likelihood of (2) can be approximated by

(6) $$\begin{array}{c} \Lambda \left(T\right)\approx pn-\left(1-p\right)k_{0}\sum_{i=1}^{n}\left[e^{-\omega (T-t_{i})}-1\right]. \end{array}$$

Combining Equations (2), (5) and (6), the total log-likelihood function can be approximated by

(7) $$\begin{array}{ccc} L(\vec{\theta }) & = & \sum_{i=1}^{n}\log\left[pn\mu_{sm}\left(t_{i}\right)+\left(1-p\right)k_{0}\sum_{j=1}^{i-1}\omega e^{-\omega \left(t_{i}-t_{j}\right)}\right]\\ & - & pn+\left(1-p\right)k_{0}\sum_{i=1}^{n}\left[e^{-\omega (T-t_{i})}-1\right]. \end{array}$$

In temporal point processes, events are assumed not to occur coincidentally, while real world data may not strictly follow this requirement. In this research, both day and month time units have been considered for model building. They are, respectively, named day-based and month-based models, i.e., the unit of arrival time for each event is an integer representing a day or month. In Lewis et al.’s research on insurgencies in Iraq, they treated the events that happened on the same day as statistically independent [7]. However, when using month as the time unit, this assumption is unreasonable, because the majority of self-exciting effects would be neglected by regarding events in the same month as independent. Based on the GTD data, it was observed that over 80% of the months in which terror attacks occur contain more than one event in all three countries, while only 20–40% of the days with terror attacks have multiple incidents in those countries. Moreover, Berrebi and Lakdawalla [6] found, in their research on Israeli data, that the risk of subsequent attacks increases the day after a terror attack and remains high for approximately eight weeks. Based on these observations, unlike the day-based model, the self-exciting effect between events in the same month is significant and non-negligible.

To ensure the interpretability of the model, we introduce an order marker for events that happened in the same month. We illustrate this idea using a toy model of 50 terror events which happened at $t_{q}$ (an arbitrary month). A unique order marker is introduced to indicate that all events occur in chronological order, and the 50 events are represented as the sequence ${\left\{t_{q},x\right\}}_{x=1,2,\cdots ,50}$. To account for the approximate exciting effect of events in the same month, we add a reward term for the order marker in λ(t) in (5). Instead of (2), we now have

(8) $$\begin{array}{ccc} \lambda (t_{q|x}) & = & pn\mu_{sm}\left(t\right)+\left(1-p\right)k_{0}\left[\sum_{t_{i}<t_{q}}\omega e^{-\omega \left(t_{q}-t_{i}\right)}+\left(x-1\right)\omega e^{-0.5\omega }\right], \end{array}$$

where $t_{q|x}$ can be regarded as $t_{q}+\left(x-1\right)\Delta t$, Δt→0. The term $\left(x-1\right)\omega e^{-0.5\omega }$ in (8) is the reward term. For events occurring in the same month, it can be seen that each event preceding the current event contributes $Ce^{-0.5\omega }$ to the intensity function, where $C=\left(1-p\right)k_{0}\omega$. The introduction of this particular reward term is intuitive, so that the triggering effect among events occurring in the same month should be greater than the triggering effect within the events occurring in previous months. Here, the triggering effect among events in the same month is taken to be a constant which is larger than $Ce^{-\omega }$. This leads to a larger contribution than the second term in the logarithm in (7), which results in a smaller *p* by MLE, indicating that a larger proportion of the process is contributed by the endogenous intensity.

**Figure 2:**
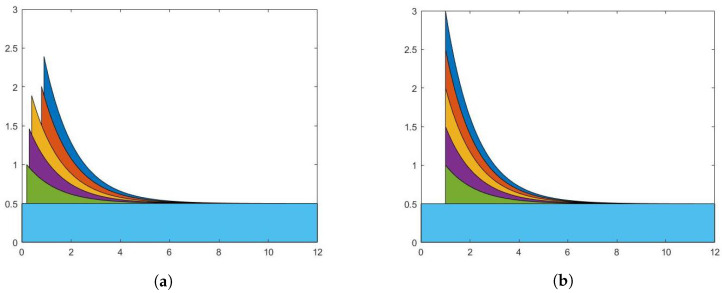
A toy example: Comparison of the conditional intensities between the rough arrival times and the natural arrival times for a simple Hawkes process with a constant background intensity during a 12-month period with 5 events occurring in the first month. (**a**) The conditional intensity of natural arrival times. (**b**) The condition intensity of rough arrival times.

The reward term described above is useful in obtaining more interpretable parameters. The integral of λ(t), which can be interpreted as the expected number of events during a certain time window based on the self-exciting model, is critical in predicting the number of terror attacks in a given year. Let $\bar{t}$ denote the vector consisting of *n* natural arrival times of events, which satisfies the strict inequality ${\bar{t}}_{1}\neq {\bar{t}}_{2}\neq \cdots {\bar{t}}_{n}$, while the corresponding arrival times in month are called “rough arrival times”. When T→∞,

$$\int_{0}^{T}\lambda \left(u|H_{t}\right)du=\int_{0}^{T}\lambda \left(u|H_{\bar{t}}\right)du,$$

where $H_{t}$ and $H_{\bar{t}}$ represent the history in rough arrival times and natural arrival times, respectively. The reason for this is that the integral value of the triggering intensity function is determined by the arrival jump and the decay rate, which are constants. With the fixed history, if *T* is sufficiently large, the self-exciting effect eventually converges to 0. A toy example comparing the conditional intensities between the rough arrival times and the natural arrival times is shown in Figure 2. It can be seen that the integral value throughout a year of the conditional intensity using the rough arrival times is nearly the same as that using the natural arrival times. More benefits of using “rough arrival times” are discussed in Section 3 and Section 4.

When exploring the best set of parameters, we chose a limited-memory version of the Broyden–Fletcher–Goldfarb–Shanno (BFGS) algorithm, which is one of the most effective quasi-Newton methods [25].

## 3. Results

The temporal patterns of terror attacks in three representative countries (the US, Turkey and the Philippines) are examined. All results are obtained using *R*. The training sets, i.e., the data from 1970 to 2017, are used as the history on which the intensity function is conditioned. To illustrate the effectiveness of the self-exciting model proposed, we compare our models with a model without self-excitation, namely when p=1. The Bayesian Information Criterion (BIC) [26] is employed as a measure to evaluate the goodness of fit.

(9) $$\begin{array}{c} \mathrm{BIC}=-2L+2\log(n)\times q, \end{array}$$

where *q* denotes the number of variables in the model.

Spanning 576 months, 2896, 4326 and 6916 terror attacks occurred in the US, Turkey and the Philippines, respectively. The information listed in Table 1 uncovers several features of the fitted models to be explained in the following paragraphs. As expected, the BIC values of the proposed models are lower than the models without the self-excitation term. It is necessary to note that the BIC values of the day-based models cannot be directly compared with those of the month-based models due to the different data formation.

The parameter $\hat{p}$ is the estimated proportion of background events. The integral of the excitation term is

$$-\left(1-p\right)k_{0}\sum_{i=1}^{n}\left[e^{-\omega (T-t_{i})}-1\right],$$

which can be approximated by $n\left(1-p\right)k_{0}$ if *T* is much larger than the arrival times of events. If the model is regarded as a Poisson cluster process, $\left(1-p\right)k_{0}$ can be interpreted as branching ratio, which gives the expected number of offspring for each event. The value of the exponential term of (2) decays with the time starting from 1, and the estimated decay rate is $\hat{\omega }$. In our model, ${\hat{\omega }}^{-1}$ captures the decay period $t_{d}$, which is the time it takes for the exponential term to decay from $e^{-d}$ to $e^{-(d-1)}$, where *d* is a non-negative integer. Hence, the smaller ω is, the longer the excitation effect of each event lasts. Lastly, the values of $\int_{0}^{T}\hat{\lambda }\left(t\right)dt$ are the expected number of events occurring during [0,T].

When comparing the estimated parameters for the same country but based on data with different units of arrival times, the values of $\hat{p}$ are relatively similar, indicating that different data resolutions have no significant effect on the estimation of the proportion of offspring and immigrants. Although the day-based models have much smaller values of $\hat{\omega }$ than those of the month-based models, the decay period in actual time given by the month-based model is longer. For example, in the US, the decay period of the day-based model is 1/0.021≈48 days, which is less than 1/0.371≈2.7 months. In the month-based model, where the arrival times of the events are approximated, the self-exciting effect of each event will emerge later and usually over a smaller number of temporal units compared with events with natural arrival times. Consequently, a longer decay period in the month-based models will result in a stronger self-exciting effect within the same period as compared with the day-based model. Moreover, the expected number of events predicted in the month-based model is even closer to the true real value compared with that given by the day-based model. This result can be attributed to the omission of specific date information at some data points, which subsequently affects the performance of the day-based model. Therefore, we conclude that the month-based model captures more of the self-exciting effect among the data.

It is very interesting to observe that the estimated values of $k_{0}$ are all around 1, which is reasonable. Recall that (1−p)n estimates the total number of offspring and $\left(1-p\right)k_{0}$ the expected number of offspring for each event. It follows that

$$\frac{(1-p)n}{n}=1-p\approx \left(1-p\right)k_{0},\;\mathrm{and}\;k_{0}\approx 1.$$

Overall, there is no conclusive evidence that the day-based model performs better than the month-based model. However, on the other hand, the month-based model is significantly more computationally efficient. For instance, the processing time of the month-based model for estimating Turkey’s parameters is 42.95 s, whereas the processing time for the day-based model is 1556.50 s (both ran on a PC with Intel(R) Core(TM) i7-11700K processor (3.6 GHz) with sufficiently large physical memory). Furthermore, the use of rough arrival times can help prevent model overfitting, which might occur with a day-based model that attempts to fit the number of events for each individual day (see the simulation performance based on both models in Section 4). Thus, the month-based model is preferable in our study.

**Figure 3:**
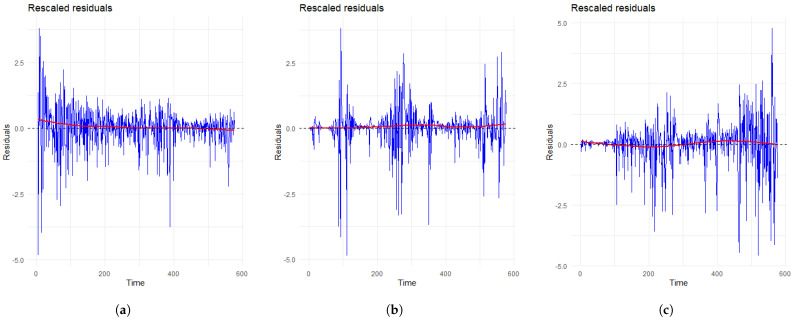
Residual plots (the residuals are scaled by their standard error): Blue: scaled residuals; red: local regression (LOESS) curve representing the overall trend of residuals; black: the reference line at zero. (**a**) Scaled residual plot for the month-based model in the US. (**b**) Scaled residual plot for the month-based model in Turkey. (**c**) Scaled residual plot for the month-based model in the Philippines.

Figure 1 shows the number of terror attacks from 1970 to 2017, as well as the estimated intensities based on the month-based models in three countries. In addition Figure 3 shows the associated residual plots for the scaled residuals. For temporal point processes, the residual analysis operates on the integral value of each time window, spanning from [0,1) to [T−1,T). For the month-based model’s context, an event’s self-exciting effect only emerges after the month it occurs. Hence, we use the average value of the integral of the conditional intensity function within [t−1,t) and [t,t+1) as the fitted value of the month *t* for the month-based model. Figure 3 shows a considerable majority of the data points have residuals oscillating around the zero line in all three countries. Despite the model’s residuals showing some larger fluctuations during periods of rapid increase, their overall tendency to hover around the zero line demonstrates that the fitting results, on average, accurately capture the key patterns in the data.

## 4. Simulation and Prediction

In this section, the simulation process based on the conditional intensity function as defined in (2) is discussed for the purpose of predictions. To avoid ambiguity, the time-stamps of the events used are months in this section. During the simulations, both the background intensity functions and parameters are estimated using the training sets.

**Figure 4:**
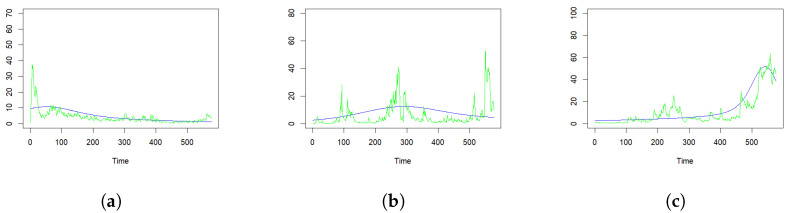
The conditional intensities of three countries with the background intensities in the same scale (month-based arrival times): The blue line is the smooth background intensity $n\mu_{sm}\left(t\right)$, while the green line is the conditional intensity λ(t). (**a**) The trend of intensities for the US data from 1970 to 2017. (**b**) The trend of intensities for the Turkey data from 1970 to 2017. (**c**) The trend of intensities for the Philippines data from 1970 to 2017.

A popular method for simulating a nonhomogeneous Poisson process with a known intensity function $\lambda_{np}\left(t\right)$ was introduced by Lewis and Shedler [27]. The method initially simulates a homogeneous Poisson process with the intensity λ that satisfies $\lambda \geq \lambda_{np}\left(t\right)$ and then stochastically rejects excess points. For a self-exciting point process, Ogata [28] proposed a modified version that only requires the local boundaries (generally defined as $\lambda (t^{+})$ for any event time *t*) of the conditional intensity function instead of a global upper bound. In the context of the model represented by Equation (2), it is not appropriate to use $\lambda (t_{i}^{+})$ as the local boundary from time $t_{i}$ to $t_{i+1}$ after an event has occurred at time $t_{i}$, due to the possibility of an increase in the smooth background intensity value between the two occurrences. In order to simulate the model based on (2), we consider the following comparatively simple conditional intensity function

(10) $$\begin{array}{c} \lambda^{*}\left(t\right):=\mu +\left(1-p\right)k_{0}\sum_{t_{i}<t}\omega e^{-\omega \left(t-t_{i}\right)}, \end{array}$$

where $\mu =\max(pn\mu_{sm}\left(t\right))$. Note that $\lambda^{*}\left(t\right)\geq \lambda \left(t\right)$, where λ(t) is given by (2), which shares the local boundaries with $\lambda^{*}\left(t\right)$. Next, for our self-exciting model, the value of the background intensity at each time point depends on all known events in the training set. Figure 4 shows that for the three countries, the background intensities do not fluctuate dramatically during short periods and represent decreasing trends at the right tail, i.e., the last few months. As a result, we simply use (3) to estimate the background intensity in 2018. In that case, the difference between the estimated time and all known events’ time increases from the 577th month to the 588th month, indicating the background intensity decreases strictly after the 576th month based on our simulations.

Algorithm 1 summarizes the steps of the thinning method for simulating the proposed model.
**Algorithm 1:** Thinning method for simulating the self-exciting process defined by (2).
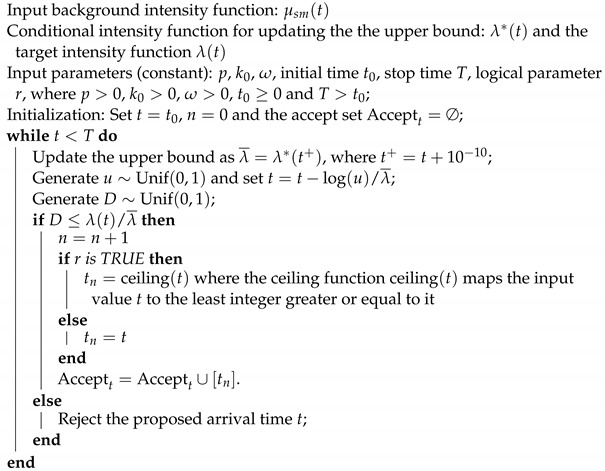


### 4.1. Direct Simulation (DS)

A general approach for prediction for point processes is to simulate the interarrival times of events in the target forecast time interval. In this paper, 500 sample paths in 2018 of each target country are simulated with the known history from the corresponding training set.

For the direct simulation method, the initial time and the stop time are set as $t_{0}=576$ and T=588, respectively ($t_{0}$ = 17,532 and *T* = 17,897 for the day-based model). In Algorithm 1, we set the logical parameter *r* to TRUE for simulating sample paths in 2018, which rounds each simulated arrival time to the smallest integer that is larger than or equal to the arrival time itself. The total number of simulated events in the target period for any simulation path is treated as a predicted value, and we set the 5% and 95% quantiles of the predicted values as the lower and upper bounds for prediction, namely prediction interval. Further, the Absolute Percentage Error (APE) [29] for the median of predicted values is calculated as the metric to evaluate the accuracy of prediction.

(11) $$\begin{array}{c} \mathrm{APE}=\frac{\left|r-p\right|}{r}, \end{array}$$

where *r* and *p* represent the real value and the predicted value, respectively. The closer APE is to 0, the more accurate the prediction result is.

Table 2 presents the actual number of terror attacks in 2018 is 67 for the US, 94 for Turkey and 601 for the Philippines. As shown in Table 3, the prediction intervals of all three day-based models are much wider than those of the month-based models. It can be seen from the median values that the month-based models tend to simulate more events than the day-based models. This finding is consistent with the discussion in Section 3. Given less computing cost, much narrower prediction ranges and smaller APEs, the month-based model appears to be the superior option for forecasting the number of terror events.

To more comprehensively assess the efficiency of the month-based model, we also employ two widely recognized data-driven models for time series forecasting, namely the Autoregressive Integrated Moving Average (ARIMA) model and the Exponential Smoothing (ES) model [30]. Both models are deployed using the R package “forecast”, which automatically selects the optimal model configurations [31]. The performance of forecasting methods is evaluated using two indicators: Root Mean Squared Error (RMSE) and Symmetric Mean Absolute Percentage Error (SMAPE), as illustrated in the following equations:

(12) $$\begin{array}{c} \mathrm{RMSE}=\sqrt{\frac{1}{m}\sum_{i=1}^{n}{\left(r_{i}-p_{i}\right)}^{2}}, \end{array}$$

(13) $$\begin{array}{c} \mathrm{SMAPE}=\frac{1}{m}\sum_{i=1}^{n}\frac{∥r_{i}-p_{i}∥}{∥r_{i}∥+∥p_{i}∥}. \end{array}$$

In these equations, ‘$r_{i}$’ and ‘$p_{i}$’ denote the actual and predicted number of events at a specified time, marked by the index ‘*i*’. Additionally, ‘*m*’ represents the count of data points used in the prediction. It is important to mention that multiple versions of the SMAPE function exist. Function (13) alters the standard SMAPE formula by dropping the multiplier of 2 in the denominator, ensuring a value range of 0% to 100% for interpretability. For both measures, the smaller the values are, the better the model performance is.

Table 4 presents a comparison of the forecasting performance of four models for three countries. While the month-based model shows a subpar performance in the US, it stands out for Turkey and the Philippines, achieving the smallest RMSE and SMAPE scores. These results re-emphasize the month-based model’s superior ability to forecast the number of terror events in these regions. In the context of the US, the self-exciting model’s performance is subpar because the Gaussian KDE causes a steady decrease in background intensities throughout the simulation period, contrasting with the fluctuating trends in the tail of the conditional intensity function (see Figure 4a).

### 4.2. Hybrid Prediction Method (HP)

Due to the uncertainty involved in simulating self-exciting processes, the prediction interval for direct simulation appears to be excessively large. Therefore, we propose a new prediction method that uses simulation and the random forest algorithm to obtain better accuracy and narrower prediction intervals.

**Figure 5:**
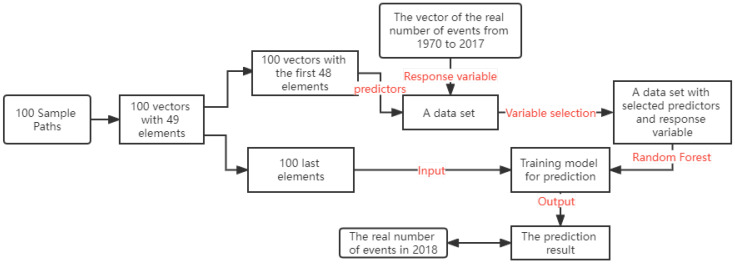
The flow chart of the hybrid prediction approach (HP).

Figure 5 presents the work flow of our proposed algorithm. Each simulation path consists of the arrival times of simulated events from 1970 to 2018. In the month-based models, $t_{0}$ and *T* in Algorithm 1 are, respectively, set as 0 and 588, while for the day-based models, *T* = 17,897. Due to the enormous computing cost of using the day-based model, in this section, we only discuss the proposed algorithm to simulate monthly arrival times.

After simulating 100 paths through 588 months, the proposed algorithm converts each sample path to a vector with 49 components by counting the number of simulated events in each 12-month interval from (0,12] to (576,588]. Hence, each component represents the number of simulated events in a year. Then, each vector is split into two parts. One consists of the first 48 components and the other the last component. Similarly, the training and test sets are also converted to vectors consisting of the number of terror attacks in each year. With these preparations, we construct a data set in which the vectors of simulated paths serve as predictors, and the vector of the actual number of occurrences serves as the response variable. Machine learning techniques, such as random forests, can then be used to construct a model for directly predicting the annual number of terror attacks.

To be consistent with the direct simulation method, the logical parameter *r* in Algorithm 1 is set as TRUE. As the prediction paths are based on the estimated background intensity function and estimated parameters from the real data, the predictor step in Figure 5 is structured and based on the idea that some simulated paths can help predict the trend of the number of events in the real data.

After the data set D with 100 predictors and a response variable is generated, a backward variable selection method called Recursive Feature Elimination (RFE) is applied to select the best fit paths. The algorithm builds a model using a large set of features and calculates an importance score for each variable included at the beginning; then, it repeatedly removes the weakest feature(s) until a proposed number of features is reached. RFE is frequently applied with random forests because it has a built-in mechanism, such as entropy [32], for measuring feature importance. Generally, the best number of predictors is unknown; therefore, recursive feature elimination with cross-validation (RFECV) [33] is applied. In REFCV, RFE determines the best subset of variables for different numbers of variables and 10-fold CV determines the best number of variables to be selected.

The algorithm applies RFECV to choose the optimal set of predictors that produces the minimum Root Mean Squared Error (RMSE) based on the random forests’ prediction outcomes. Finally, the random forest algorithm is used to create the training model for predicting the number of events in a year. With different random seeds, the selected factors and the outcome of the prediction can vary. To produce a more reliable result, 100 random forest models are constructed using 100 different random seeds. Similar to the direct simulation method, the empirical prediction interval of the 100 random forest models is specified by their 5% and 95% quantiles.

Table 5 compares the performance between the Direct Simulation method (DS) and the Hybrid Method (HM) based on the full path simulation and the random forest algorithm in predicting the number of events for 2018. In this table, we provide the first quartile (Q1), the median (now expressed as Q2 for consistency) and the third quartile (Q3) of prediction values for a thorough comparison. While HM displays a slightly larger APE at the median (APE${}_{Q2}$) compared with DS, it achieves superior performance in terms of APE${}_{Q1}$ and APE${}_{Q3}$. This superiority indicates that HM offers a more precise prediction interval. Furthermore, the actual number of terror events that occurred in 2018 for the US (67), Turkey (94) and the Philippines (601) all fall within the prediction intervals generated by HM. This method also demonstrates robustness as evidenced by its narrower prediction intervals compared with those generated by DS.

In order to further verify the prediction performance of our proposed model, we calculate APEs for two popular methods—ARIMA and ES—as well as compare them with the APEs from HM and DS to calculate Percentage of Superior APE Predictions (PSAP). A greater value of PSAP signifies a larger share of predictions with lower APEs than the compared method, thus implying superior predictive performance.

(14) $$\mathrm{PSAP}=\frac{1}{N}\sum_{i=1}^{N}I\left({\mathrm{APE}}_{i}<{\mathrm{APE}}_{RM}\right),$$

where ${\mathrm{APE}}_{1}$ to ${\mathrm{APE}}_{N}$ represents the APE from the method under consideration, obtained over *N* runs. ${\mathrm{APE}}_{RM}$ is the APE for the reference method. The function *I* is an indicator function, which equals 1 when the condition ${\mathrm{APE}}_{i}<{\mathrm{APE}}_{RM}$ is met, and 0 otherwise.

Table 6 lists the PSAP values for DS and HM against ARIMA and ES. Evidently, the HM consistently demonstrates a higher PSAP across all three countries, indicating its superior prediction performance over DS when compared with standard models such as ARIMA and ES. Despite both DS and HM having a high proportion of accurate predictions that outperform ARIMA and ES, challenges persist when applied to the US data, as indicated by the HM’s PSAP values falling below 50% when compared with ARIMA. This suggests that in order to improve the model’s prediction performance for the US data, it may be helpful to employ a more suitable background intensity function.

In summary, for the three countries studied, HM yields prediction intervals that are both narrower and more accurate. Thus, HM is capable of estimating the number of terror attacks with a high precision when compared with the DS approach.

## 5. Discussion and Conclusions

In this paper, we introduce an innovative self-exciting process model using the “rough arrival times” instead of the “natural arrival times”, thereby effectively streamlining both parameter estimation and model simulation. To consider the self-exciting effect among events occurring within the same month in our model, we introduce two new tuning parameters: the order mark and the reward term. Consequently, our proposed month-based model significantly reduces the computational burden compared with the day-based model, while offering more precise estimates of the number of terror events in the simulation for year 2018. Furthermore, it provides insights on how self-exciting point processes can be effectively applied to the data without very fine-grained time-stamps such as days.

The proposed self-exciting point process also has advantages in practical applications, largely due to the interpretability of its parameters. For instance, if the background intensity is the same, then the self-excitation term can vary and reflect the efficacy of local counter-terrorism efforts. In our case studies, the estimated value of *p*, the proportional contribution of the background intensity, is found to be small, indicating the largest contribution comes from the self-excitation term. This aligns with the SSSL model [3], which treats terrorism as a learned behavior. Moreover, we propose a hybrid prediction approach that integrates simulation and random forests to create a short-term forecasting strategy for the frequency of terror attacks. This tool can be very meaningful for policymakers in assessing the risk of terrorism, thereby strategically making decisions regarding more effective prevention measures.

For future research, the proposed model can include spacial information, as terror events often are geographically clustered. The spatial information can be incorporated into both the background intensity as well as the self-excitation term, evolving the model into a multidimensional variant. A spatiotemporal self-exciting point process could potentially capture the dynamic hot-spots of terrorism in both space and time, allowing for a more comprehensive analysis of cross country self-exciting effects for terror data. Moreover, to improve our model’s predictive capabilities, the development of a more appropriate background intensity function is expected. Furthermore, the simulation method employed in this paper is one type of Monte Carlo simulation method. When using multivariate self-exciting processes, simulation methods such as the thinning method are costly and complicated. In this regard, a Deep Point Process, which combines deep neural networks and point processes, offers potential solutions to these challenges [34].

## Figures and Tables

**Table 1:** Parameter estimates, the integral of the intensity functions and BIC values of models for the US, Turkey and the Philippines based on Equation (2), where *d* and *m* represent unit day and unit month, respectively. NS BIC is the BIC for the model without self-excitation.

Country/Unit	$\hat{p}$	${\hat{k}}_{0}$	$\hat{\omega }$	$\int_{0}^{T}\hat{\lambda }\left(t\right)\mathrm{dt}$	BIC	NS BIC
US/d	0.174	1.002	0.021	2862.036	13,981.490	15,265.980
US/m	0.103	1.004	0.371	2896.001	−5634.409	−4409.704
Turkey/d	0.097	1.001	0.055	4300.921	13,955.890	20,453.320
Turkey/m	0.059	1.006	0.546	4326.003	−14,900.880	−9026.814
Philippines/d	0.163	1.010	0.028	6882.001	17,082.750	19,481.090
Philippines/m	0.148	1.029	0.336	6915.998	−29,826.880	−27,503.260

**Table 2:** The actual monthly count of terror attacks in three countries throughout the year 2018.

Country	January	Feburary	March	April	May	June	July	August	September	October	November	December
US	2	1	6	3	6	1	6	5	4	24	8	1
Turkey	17	15	13	9	7	12	5	4	3	5	3	1
Philippines	55	38	43	61	69	49	58	57	38	41	39	53

**Table 3:** Prediction intervals (PI); prediction medians with their corresponding APEs based on simulation paths of both day-based and month-based models.

Country/Unit	PI	Median	APE
US/d	[4,52]	18	73.1%
US/m	[17,66]	36	46.3%
Turkey/d	[9142]	38.5	59.0%
Turkey/m	[59,166]	106	12.8%
Philippines/d	[385,697]	529.5	11.9%
Philippines/m	[527,724]	612.5	2.0%

**Table 4:** Performance comparison of forecasting models for the US, Turkey and the Philippines: the day-based model, the month-based model, ARIMA and ES. The metrics are computed from median counts of events in each month across 500 simulated paths.

Country	Model	RMSE	SMAPE
US	Day-based	7.47	43.43%
US	Month-based	6.86	37.54%
US	ARIMA	6.01	34.46%
US	ES	6.20	33.62%
Turkey	Day-based	6.20	47.87%
Turkey	Month-based	4.09	27.22%
Turkey	ARIMA	12.83	46.79%
Turkey	ES	5.49	32.5%
Philippines	Day-based	11.36	9.04%
Philippines	Month-based	9.94	8.88%
Philippines	ARIMA	11.51	9.37%
Philippines	ES	11.51	9.37%

**Table 5:** Prediction intervals (PI) for Q1, Q2 and Q3 with their corresponding APEs based on two different methods: DS represents direct simulation referred in Section 4.1, while HM represents the hybrid method and is based on the data set D.

Country	Method	PI	Q1	Q2	Q3	APE${}_{Q1}$	APE${}_{Q2}$	APE${}_{Q3}$
US	DS	[17,66]	26	36	46	61.1%	46.3%	31.3%
US	HM	[41,84]	45.75	52	62.25	31.7%	22.4%	7.1%
Turkey	DS	[59,166]	86	106	130	8.5%	12.8%	38.3%
Turkey	HM	[76,119]	86.75	99	108.25	7.7%	5.3%	15.1%
Philippines	DS	[527,724]	580	612.5	653	3.5%	2.0%	8.7%
Philippines	HM	[595,640]	602	616	627	0.7%	2.5%	4.3%

**Table 6:** APEs of the ARIMA and ES methods and the Percentage of Superior APE Predictions (PSAP) comparing DS and HM.

Country	Reference Method	APE	PSAP${}_{\mathrm{DS}}$	PSAP${}_{\mathrm{HM}}$
US	ARIMA	20.9%	12.8%	32%
US	ES	28.4%	22.6%	65%
Turkey	ARIMA	140.4%	99.8%	100%
Turkey	ES	27.7%	54.4%	94%
Philippines	ARIMA	11.8%	77%	100%
Philippines	ES	11.8%	77%	100%

## Data Availability

The Global Terrorism Database, which is funded by the University of Maryland, provided the data for this study. The data was accessed on 3 May 2020 and can be requested through https://www.start.umd.edu/gtd/.

## Abbreviations

The following abbreviations are used in this manuscript:

RDT	Relative Deprivation Theory
GST	General Strain Theory
SLT	Social Learning Theory
SSSL	Social Structure Social Learning
GTD	Global Terrorism Database
KDE	Kernel Density Estimation
KNN	K-Nearest Neighbor
MLE	Maximum Likelihood Estimation
BFGS	Broyden–Fletcher–Goldfarb–Shanno Algorithm
APE	Absolute Percentage Error
PI	Prediction Interval
RMSE	Root Mean Squared Error
SMAPE	Symmetric Mean Absolute Percentage Error
ARIMA	Autoregressive Integrated Moving Average
ES	Exponential Smoothing
RFE	Recursive Feature Elimination
RFECV	Recursive Feature Elimination with Cross-Validation
DS	Direct Simulation
RF	Random Forest
HM	Hybrid Method
PSAP	Percentage of Superior APE Prediction
